# GP73 is down-regulated in gastric cancer and associated with tumor differentiation

**DOI:** 10.1186/1477-7819-11-132

**Published:** 2013-06-07

**Authors:** Le-Gao Chen, Hui-Ju Wang, Hai-bo Yao, Tian-Pei Guan, Fang Wu, Xu-Jun He, Ying-Yu Ma, Hou-Quan Tao, Zai-Yuan Ye

**Affiliations:** 1 Key Laboratory of Gastroenterology of Zhejiang Province, Zhejiang Provincial People’s Hospital, Hangzhou 310014, Zhejiang, China

**Keywords:** GP73, Gastric cancer, Tumor differentiation

## Abstract

**Background:**

Golgi protein 73 (GP73) is a type II Golgi transmembrane protein. It is over-expressed in several cancers, including hepatocellular carcinomas, bile duct carcinomas, lung cancer and prostate cancer. However, there are few reports of GP73 in gastric cancer. This study is aimed at investigating the expression of GP73 and its relationship with clinical pathological characters in gastric cancer.

**Methods:**

GP73 mRNA level was determined by quantitative real-time RT-PCR in 41 pairs of matched gastric tumorous tissues and adjacent non-tumorous mucosal tissues. Western blotting was also performed to detect the GP73 protein level. GP73 protein expression was analyzed by immunohistochemistry in 52 clinically characterized gastric cancer patients and 10 non-tumorous gastric mucosal tissue controls.

**Results:**

The mRNA and protein level of GP73 were significantly down-regulated in gastric tumorous tissues compared with the non-tumorous mucosal tissues. In non-tumorous mucosa, strong diffuse cytoplasmic staining can be seen in cells located at the surface of the glandular and foveolar compartment; while in tumorous tissues, the staining was much weaker or even absent, and mainly in a semi-granular dot-like staining pattern. The expression level of GP73 protein was associated with patients’ gender and tumor differentiation.

**Conclusions:**

GP73 was normally expressed in non-tumorous gastric mucosa and down-regulated in gastric cancer. Its expression in gastric cancer was correlated with tumor differentiation.

## Background

GP73, also known as GOLM1 and GOLPH2, is a type II Golgi transmembrane protein. Structurally, GP73 contains a short N-terminal cytoplasmic domain, a transmembrane domain and a larger luminal C-terminal domain which is composed of a coiled-coil domain and an acid tail. The luminal C-terminal domain is the major function domain of GP73 [1-3]. Mice with a severe truncation of the GP73 C-terminus showed decreased survival and severe epithelial abnormalities of the kidney and the liver [4]. The coiled-coil domain could interact with both precursor and mature sCLU, indicating that GP73 might assist in the post-translational modification, transportation and secretion of sCLU [5]. A proproteinconvertase recognition site R^52^VRR^55^ is located within the C-terminal ectodomain; PC-mediated cleavage of GP73 transforms the intracellular, Golgi-localized GP73 to a soluble, secretory protein [6]. However, the function of GP73 is still unclear.

GP73 was first identified from a cDNA library derived from the liver of a patient with adult giant-cell hepatitis. It is considered an epithelial cell-specific protein, which is highly expressed in the colon, stomach, prostate and trachea in normal, healthy persons [1]. Liver disease, such as virus infection and cirrhosis, could up-regulate the expression of GP73 in hepatocytes [1,7,8]. Recent studies showed that GP73 was over-expressed in several cancers, such as hepatocellular carcinomas [9-11], bile duct carcinomas [11], lung adenocarcinomas [12], prostate cancer [13,14] and seminomas [15]. The expression of GP73 in renal cancer is much more comprehensive. It was reported as down-regulated in the majority of clear cell renal cell cancers (RCCs), but highly-expressed in papillary and chromophobe renal carcinomas [16]. Serum GP73 has also been found elevated in hepatocellular carcinomas, bile duct carcinomas and lung adenocarcinomas. It even showed an advantage over α-fetoprotein (AFP) for diagnosing early hepatocellular carcinomas [17,18]. Also, mRNA expression of GP73 in urine outperformed serum PSA in detecting prostate cancer [13].

Gastric cancer is one of the most common cancers and the second leading cause of cancer-related death worldwide. It has particularly high frequencies in China [19]. Until now, the etiological factors and pathogenesis of gastric cancer have not been fully understood. In this study, we aim to investigate the expression of GP73 and its relationship with clinical pathological characters in gastric cancer by measuring the mRNA and protein expression level of GP73 in gastric tumorous and adjacent non-tumorous mucosal tissues.

## Methods

### Materials and patients

Forty-one pairs of matched gastric tumorous and adjacent non-tumorous mucosal tissues (>5cm from the edge of the tumor)were obtained from patients with primary gastric cancer at the Zhejiang Provincial People’s Hospital, China from January 2011 to December 2012. The characteristics of 41 GCsare shown in Table 1. After surgical removal, tissues were frozen immediately in liquid nitrogen and stored at −80°C until use.

**Table 1:** Summary of clinicopathologic characteristics of gastric cancer patients

**Clinical parameters**	**N (%)**
**Gender**	
Men	27(65.9)
Women	14(34.1)
Size	23(56.1)
<20	18(43.9)
≧20	
**Histologic differentiation**	
Well	3(7.3)
Moderately	17(41.5)
Poorly	21(51.2)
**Lymphatic metastasis**	
No	11(26.8)
Yes	30(73.2)
**Distant metastasis**	
No	34(82.9)
Yes	7(17.1 )
**TNM stage**	
I	8(19.5)
II	17(41.5)
III	10(24.4)
IV	6(14.6)

Fifty-two paraffin specimens of gastric cancer (GC) tissue (43 males, 9 females) were acquired from Zhejiang Provincial People’s Hospital, collected from 2010 to 2011. All cases of gastric cancer were classified according to the WHO classification and staged by means of the pTNM system [20]. The cases consisted of 22 patientsaged<60 years, 30 patients aged ≥60years; 20 patients with the tumor size <5cm, 32 patients with the tumor size ≥5cm; 22 patients with moderately differentiated cancer, 30 patients with poorly differentiated cancer; 14 patients with T1 or T2 wall invasion, 38 patients with T3 or T4 wall invasion; 39 patients with lymph node metastasis and 13 patients without lymph node metastasis; 10 patients with distant metastasis and 42 patients with no distant metastasis. The numbers of patients classified by TNM stage were as follows: 18 were at TNM stage I or II; 34 at TNM stage III or IV. The patients were between 32 and 84 years of age, and there was no radiotherapy or chemotherapy prior to the operation. Specimens were fixed by formalin and embedded in paraffin. Tennormal tissue specimens of the stomach from patients without malignant tumors were also obtained by endoscopy as the control group.

All patients provided informed consent for the use of their tissues before surgery. The use of all specimens was approved by the ethics committee of Zhejiang Provincial People’s Hospital.

### Quantitative real-time PCR

qRT-PCR was performed to determine the mRNA level of GP73. Briefly, total RNA was extracted from 40 pairs of matched gastric tumorous and adjacent non-tumorous mucosal tissue specimens, using Trizol (Invitrogen, Camarillo, USA) according to the manufacturer’s instructions. cDNA synthesis was carried out with the PrimeScript 1st Strand cDNA Synthesis kit (Takara,DaLian, China), using 1 μg of total RNA as the template and OligodT primer under 65°C, 5 minutes, 42°C, 60 minutes and 70°C, 10 minutes of reverse transcription. The resulting cDNA was amplified by qPCR using specific primers with SYBR Premix Ex Taq (Takara, DaLian, China). GAPDH was used as an internal control. Primers for GP73 were 5′-GCAAAGCAACATCTTCCCTA-3′ (sense) and 5′-CCACAACAAACTTGCCCTC-3′ (antisense). Primers for GAPDH were 5′- TGAAGGTCGGAGTCAACGG-3′ (sense) and 5′- CTGGAAGATGGTGATGGGATT-3′ (antisense). PCR parameters were as follows: 95°C for 5 minutes, followed by 40 cycles of 95°C for 10s, 60°C for 20s and 72°C for 20s. At the end of the PCR cycles, melting curve analysis was performed. The relative expression of GP73 to GAPDH was calculated using 2^-∆CT^ method.

### Western blotting

Western blotting was performed to investigate GP73 protein level in 5 of 41 pairs of gastric tumorous tissue and adjacent non-tumorous mucosal tissue. Protein was isolated from tissues samples following the RNA extraction. And approximately 30 μg of protein was separated on 10% polyacrylamide gel for two hours. After being transferred to a polyvinylidenedifluoride (PVDF) membrane (GE Healthcare, Fairfield, Connecticut, USA), the samples were probed with primary antibodies against GP73 (1:2,000, Sigma, St. Louis, MO, USA) and β-actin (1:5,000, Sigma, USA) at 4°Covernight. After incubating with secondary antibody (1:5,000, CapitalBio, Beijing, China) for 2h, the membranes were treated with electrochemiluminescence (ECL) reagent (Generay, Shanghai, China) and exposed to autoradiographicfilms.

### Immunohistochemical staining

Immunohistochemical staining was performed by the standard method. Briefly, 52 cases of paraffin-embedded gastric tumors tissues and 10 cases of adjacent non-tumorous mucosal tissue controls were cut at 5 μm thick and placed on microscopic slides and dried in a 60°C oven for 2h. Then, the sections were de-paraffinized in xylene, rehydrated using a gradient of ethanol concentrations, microwaved in 10 mM citrate buffer for 15 minutes to retrieve antigen, blocked with 3% hydrogen peroxide for 10 minutes to inhibit endogenous peroxidase activity and incubated with 10% goat non-immune serum for 20 minutes to reduce background non-specific staining. After that, the sections were incubated with the rabbit anti-GP73 polyclonal antibody (Abcam, Cambridge, UK) (1:100 dilution) at 4°C overnight, then incubated with biotin-labeled secondary antibody (Invitrogen,USA) at room temperature for 20 minutes, followed by incubation with HRP-conjugated streptavidin (Invitrogen, USA) at room temperature for 20 minutes. Then, color development was performed with a DAB Substrate Kit (Dako, Carlsbad, USA). Finally, the sections were counterstained with hematoxylin, dehydrated, cleared and mounted.

### Evaluation of the immunohistochemicalstainings

The immunohistochemicalstainings of GP73 were scored by two pathologists independently, based on the intensity and the proportion of positively stained cells. Staining intensity was evaluated with a four-tiered grading system: 0 = negative, 1= weak, 2 = moderate and 3 = strong. The percentage of positive cells were scored as follows: 0 for no cell stained, 1 for 1% to 25% of cells stained, 2 for 26 to 50% of cells stained, 3 for 51 to 75% of cells stained and 4 for more than 75% of cells stained. Scores for intensity and percentage of positive cells were multiplied. Scores ≤3 was used to define tumors with low GP73 expression and scores ≥4 with high GP73 expression.

### Statistical analysis

Statistical analysis was performed using SPSS software 13.0 (SPSS, Inc., Chicago, IL, USA). The paired-samples t-test was used to analyze the differences of GP73 mRNA expression between tumor and non-tumorous tissues. Chi-square test was applied to assess the statistical significance of the associations between GP73 expression and clinicopathological parameters. *P*<0.05 was considered statistically significant.

## Results

### GP73 expression level in gastric tumor and non-tumorous tissue

**Figure 1:**
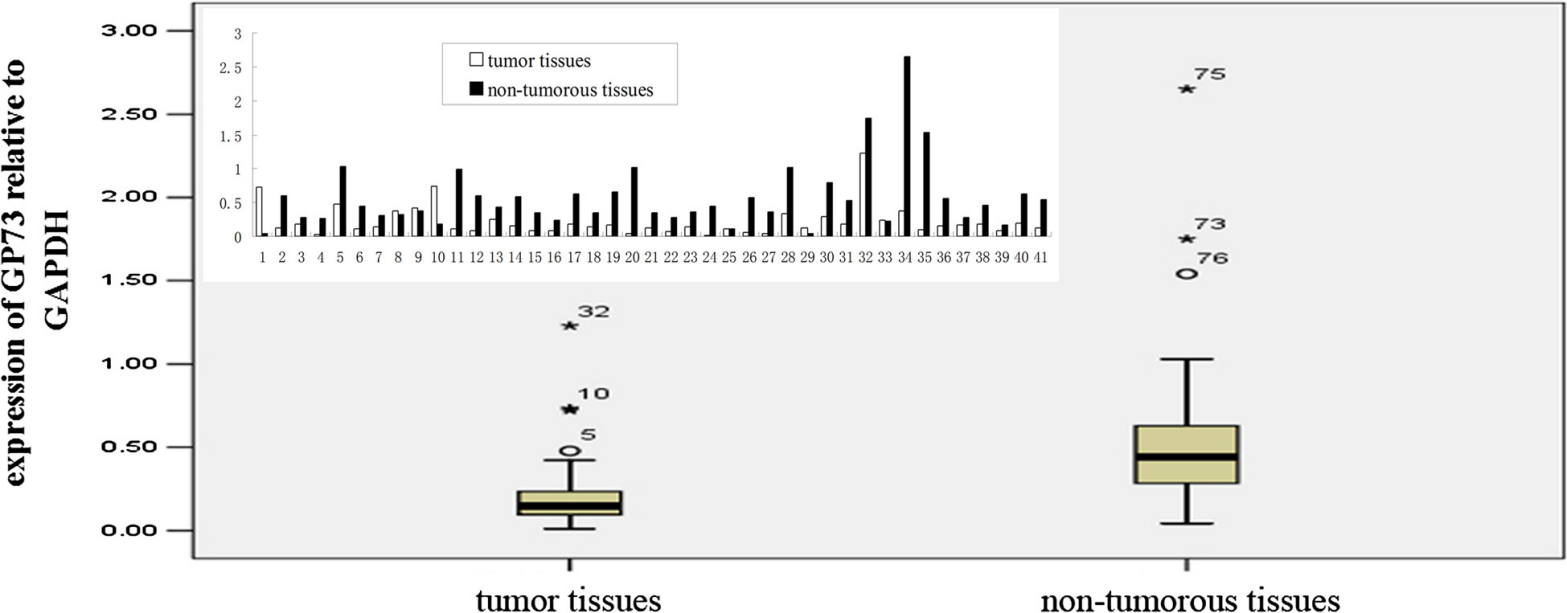
GP73 mRNA expression in gastric tumorous tissues and adjacent non-tumorous mucosal tissues.

**Figure 2:**
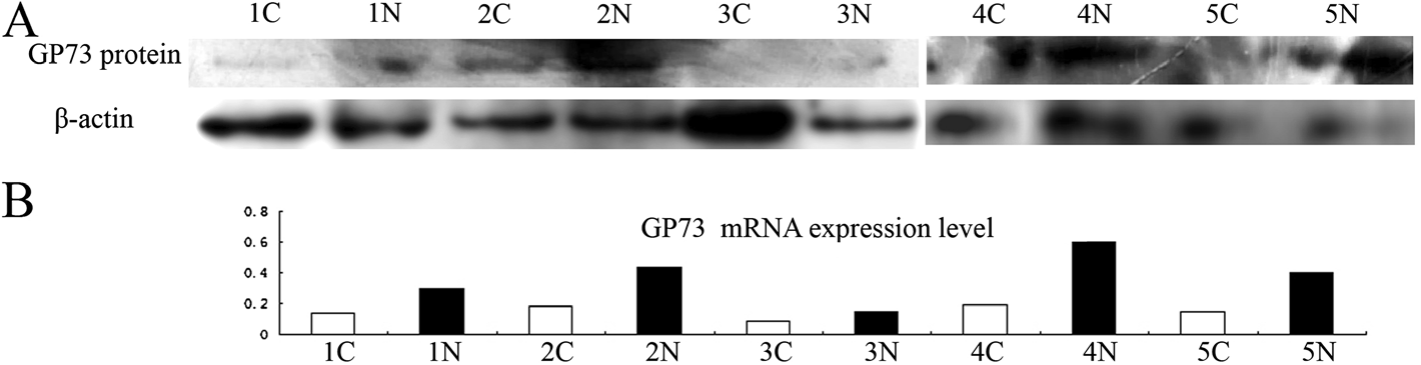
**GP73 protein level in gastric tumorous samples and adjacent non-tumorous mucosal samples. A**) GP73 protein level in gastric tumorous samples and adjacent non-tumorous mucosal samples. **B**) GP73 mRNA level in the same gastric tumorous samples and adjacent non-tumorous mucosal samples. **C**) gastric cancer; N: normal).

GP73 mRNA expression was significantly decreased in gastric tumor tissues compared to the adjacent non-tumorous tissues (33/41; 80.5%). The expression levels of GP73 relative to GAPDH were much lower in gastric tumorous tissues (0.259 ± 0.308) than that in non-tumorous mucosal tissues (0.584 ± 0.523; *P*<0.01; Figure 1). Consistent with RT-qPCR, GP73 protein level in gastric tumor samples was significantly lower than that of non-tumorous samples, which was tested by Western blotting (Figure 2).

### Correlation between GP73 protein expression and clinicopathologic parameters

**Figure 3:**
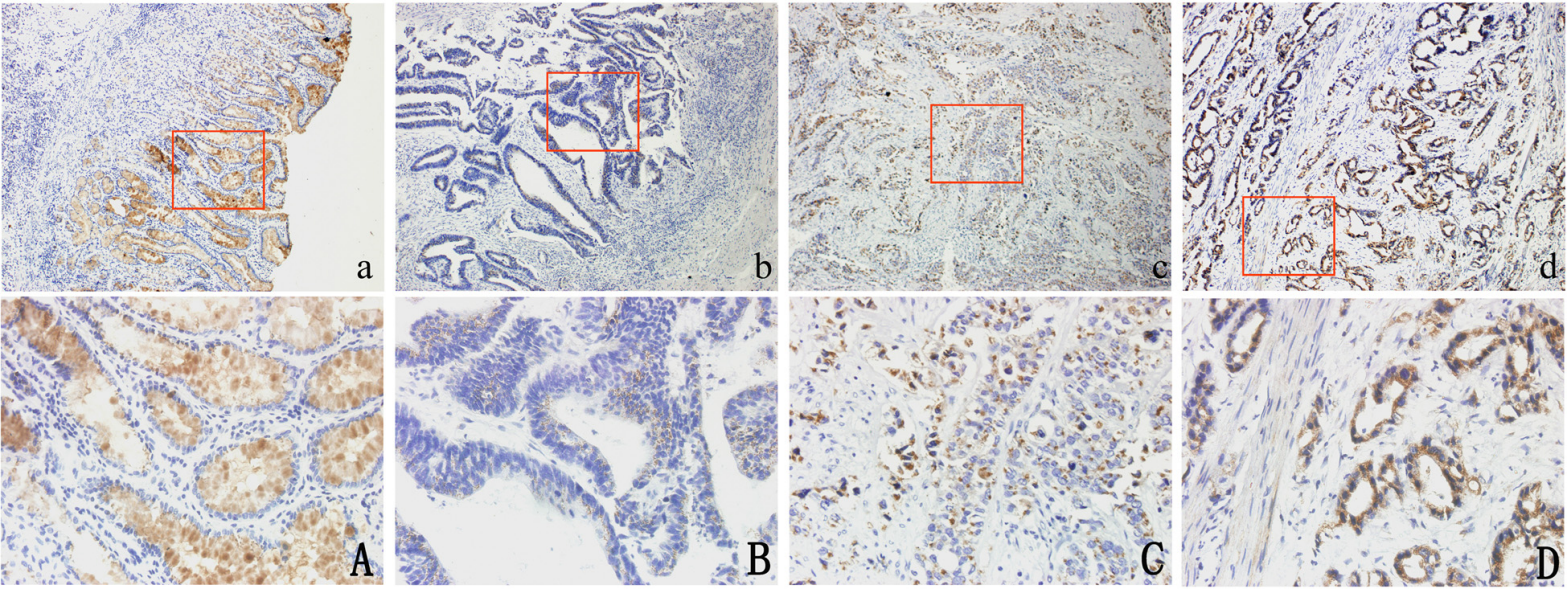
**GP73 protein expression in gastric tumorous tissues and adjacent non**-**tumorous mucosal tissues. A**) Strong expression of GP73 in non-tumorous tissues. **B**) Weak expression of GP73 in gastric tumorous tissues. **C**) Moderate expression of GP73 in gastric tumorous tissues. **D**) Strong expression of GP73 in gastric tumorous tissues. **a**,**b**,**c**,**d**: original magnification 100×. A,B,C,D: original magnification 200×.

IHC showed that GP73 was highly expressed in adjacent non-tumorous mucosal tissue, but decreased or even absent in gastric tumorous tissues. In non-tumorous mucosa, strong diffuse cytoplasmic staining could be seen in cells located at the surface of the glandular and foveolar compartment (Figure 3A). In tumor tissues, GP73 expression was mainly expressed in a distinct semi-granular dot-like staining pattern (Figure 3B, C). Only a few gastric tumor tissues were found with strong expression of GP73 (Figure 3D).

The correlation between expression of GP73 protein and clinical variables is shown in Table 2. The GP73 expression level was significantly related to the gender of patients (*P* = 0.027) and differentiation (*P* = 0.049). The expression of GP73 in poorly differentiated gastric tumors was much lower than that in moderately differentiated gastric tumors. A total of 21 out of 30 poorly differentiated gastric tumor cases showed low GP73 expression, while in moderately differentiated gastric tumor cases it was only 9/22. Also, the expression of GP73 in male patients was down-regulated more frequently than that in female patients. There was no significant correlation between GP73 expression and other clinicopathologic parameters.

**Table 2:** Relationship of GP73 expression with pathological parameters of tumor

**Clinical parameters**	**GOLPH2**		***P***-**value**
	**Low**	**High**	
Total number	30	22	
Gender			0.027
Male	28	15	
Female	2	7	
Age (years)			0.456
<60	12	10	
≥60	18	12	
Tumor size (cm^2^)			1.000
<20	14	6	
≥20	16	16	
Differentiation			0.049
Moderately differentiated	9	13	
Poorly differentiated	21	9	
Stage			0.390
I+II	12	6	
III+IV	18	16	
Depth of wall invasion			1.000
T1+T2	8	6	
T3+T4	22	16	
Lymphatic metastasis			0.757
No	7	6	
Yes	23	16	
Distant metastasis			0.725
No	25	17	
Yes	5	5	

## Discussion

Recently, several studies showed that GP73 is over-expressed in some cancers, including hepatocellular carcinomas, bile duct carcinomas, lung cancer, prostate cancer and seminomas [9-15]. In hepatocellular carcinomas, high-expression of GP73 was associated with tumor size, differentiation, grade and vein invasion [7-9]. In bile duct carcinomas, GP73 expression correlated with patient age and survival [11]. In lung cancer, GP73 expression was associated with tumor histology and patient gender; its expression in adenocarcinoma was significantly higher than in other types of lung cancer [12]. However, no correlation with clinicopathological parameters has been found in prostate cancer and seminomas [13,15]. GP73 also has been found down-regulated in clear cell RCCs, which is the most common histological subtype of renal cell cancer, while its expression in papillary and chromophobe RCC was strong [18]. In the present study, we found that both GP73 mRNA and protein levels were highly expressed in non-tumorous gastric mucosas, which were opposite to most previous studies in other cancers.

The knowledge of GP73’s function still remains limited. So far, we know that GP73 is essential for liver and kidney to maintain normal function and appearance. It is also known that GP73 may function in assisting in protein transportation and secretion. One such protein, sCLU, has already been identified. sCLU could prevent apoptosis and is over-expressed in various cancers. GP73 could interact with sCLU through coiled-coil domain and assist transportation and secretion of sCLU, which may explain the association between high expressed GP73 and the aggressive behavior of hepatocellular carcinomas [3]. In hepatocellular carcinoma (HCC), the over-expression of GP73 was not only detected in cancer tissues but also in patients’ serum, which indicated the significant increase in GP73 levels would provide a marker for early detection. Some reports even stated that GP73 is a better marker than AFP for diagnosing HCC [10,11,18,21]. However, what is the function of GP73 in stomach and why would it be down-regulated in gastric cancer? This is still unclear. GP73 is an epithelial cell-specific protein and highly expressed in numerous normal human tissues, especially in the colon and stomach. It is also predominately expressed in the small intestine, colon and stomach of mice [22]. Consistent with these studies, we also found that GP73 was highly expressed by cells located at the surface of the glandular and foveolar compartment of normal human gastric mucosa. Since the highest expression of GP73 is in epithelial cells of digestive organs, it is reasonable to presume that it may play some important, unknown roles in the digestive system.

We also found the expression of GP73 was associated with tumor differentiation. The expression of GP73 in poorly differentiated gastric tumors was much lower than that in moderately differentiated gastric tumors. It was reported that knock-down of GP73 in HepG2.2.15 cells could cause a reduction in the surface area of the Golgi complex. Since the size and development of the Golgi complex are positively correlated with tumor differentiation, some researchers hypothesized that GP73 expression might be associated with maintaining the structural integrity of the Golgi complex during oncogenesis [23,24]. Recent studies demonstrated that loss of GP73 caused up-regulation of WT1, which may promote glomus formation and inhibit pronephric tubule differentiation. It suggested that GP73 plays an important role in pronephros development and differentiation [25]. In hepatocellular carcinoma, GP73 protein was strongly associated with tumor size, vein invasion and tumor differentiation, suggesting GP73 expression is correlated with the aggressive behavior of cancer [10]. Moreover, the expression of GP73 was also associated with patient gender. Male patients showed low GP73 expression more frequently than female patients. A similar phenomenon has also been reported in lung cancer. Female patients had a higher expression of GP73 than male patients [12]. It is also reported that phenotypic changes observed in mice with truncated GP73 C-terminus are gender-dependent [4]. One explanation is that GP73 mRNA is regulated by estrogens and calcitriol [26]. Our work again confirmed the hypothesis that GP73 expression is under hormonal control.

## Conclusion

In conclusion, we found that both the GP73 mRNA and protein level was significantly down-regulated in gastric tumorous tissues compared to the non-tumorous mucosa, and the expression of GP73 was associated with tumor differentiation and patient gender. Our further work will focus on investigating the function and the regulation mechanism of GP73 in the stomach.

## Abbreviations

AFP: α-fetoprotein; ECL: Electrochemiluminescence; GC: Gastric cancer; GP73: Golgi protein 73; HCC: Hepatocellular carcinoma; PSA: Prostate specific antigen; PVDF: Polyvinylidenedifluoride; RCC: Renal cell cancer.

## Competing interests

The authors declare that they have no competing interests.

## Authors’ contributions

LGC and HJW were involved in the design of the study, performed the qRT-PCR, Western bolting and immunohistochemical staining analysis, and drafted the manuscript. ZYY and HQT were involved in the design of the study and supervised the study. HBY, FW and TPG collected data and helped to draft the manuscript. XJH and YYM provided general support and helped to draft the manuscript. All authors read and approved the final manuscript.
